# Evaluating the potential of phytoextraction on waste-to-energy bottom ash—a review

**DOI:** 10.1007/s11356-025-36399-z

**Published:** 2025-04-14

**Authors:** Karin Karlfeldt Fedje, Sofia Sjöstedt, Ann-Margret Strömvall

**Affiliations:** 1 Department of Architecture and Civil Engineering, Water Environment Technology, Chalmers University of Technology, SE- 412 96 Gothenburg, Sweden; 2 Recycling and Waste Management, Renova AB, Box 156, SE- 401 22 Gothenburg, Sweden

**Keywords:** Phytoextraction, Waste-to-energy incineration bottom ash, Metal recycling, *Sesbania drummondii*, *Salix viminalis*, *Salix alba*

## Abstract

Waste-to-energy produces district heating and electricity and generates bottom ash. This ash contains valuable chemically bound metals and current methods for extracting them face significant challenges, prompting the need for alternative methods such as phytoextraction. This review evaluates the potential of using phytoextraction on sorted and aged bottom ash to recover metals and enhance the usability of the MIBA residue in new applications. The focus is on the minor elements Cu, Zn, Pb, Ni and Co. A list of suitable terrestrial plants is suggested based on their ability to grow in the Nordic climate and the presence of the metals of interest. A further evaluation using qualitative multicriteria analysis (MCA) based on selected criteria, i.e. biomass, extraction capacity, metal diversity, perennial or annual growth, and accumulation above ground or in roots was performed. More than 100 different plants were reviewed for their suitability for MIBA phytoextraction, with 13 plants identified as the most promising. Among the selected plants, *Sesbania drummondii* scored the highest, followed by *Salix alba* and *Salix viminalis*. All these plants are perennial and can extract multiple metals; Salix exhibits lower to moderate extraction efficiency but compensates for this with high biomass and rapid growth compared to other plants with higher extraction capacity. In conclusion, the study shows the potential use of phytoextraction as a method to treat MIBA. However, further cultivation experiments are necessary to evaluate its efficiency. This review provides valuable information for designing such research.

**Graphical abstract:**

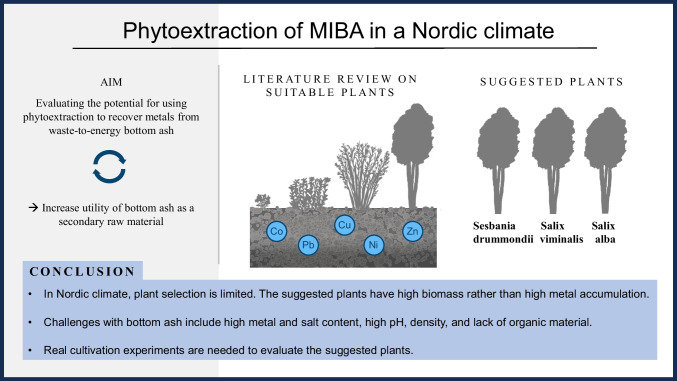

**Supplementary Information:**

The online version contains supplementary material available at 10.1007/s11356-025-36399-z.

## Introduction

Today’s society is characterised by advanced technological innovations that are critical in creating a sustainable future. The rising deployment of clean energy technologies as part of energy transitions will cause an increase in metal and mineral demand (Hund et al. 2023; International Energy Agency 2021). The critical elements for green technologies include Co, Cr, Cu, Ni, Li, Zn, platinum group metals and rare earth elements. Relying only on mining as a supply to meet these demands is linked to several environmental impacts including high energy consumption (Holmberg et al. 2017; Allen 2021), CO_2_ emissions (Norgate et al. 2007) and acidification and ecotoxicological effects in both marine and terrestrial environments (Tao et al. 2019; Zhang et al. 2021). Consequently, the recycling of metals and the use of secondary raw materials must increase. One such material is ash from waste-to-energy (WtE).

Waste-to-energy is an important waste management technology that not only degrades organic pollutants and reduces the volume and mass but also generates district heating and electricity (Johansson et al. 2014; Neuwahl et al. 2019). Waste generation as well as WtE is increasing within the EU, which leads to a higher generation of ash, in which the metals present in the waste are enriched (Eurostat 2023). Annually, about 1 Mt (Blasenbauer et al. 2020) of bottom ash (BA) is produced in Sweden. The corresponding value in the EU, including Norway and Switzerland, was almost 18 Mt in 2019 (Blasenbauer et al. 2020). Pure metal pieces are recycled from the BA and reused in society, while chemically bound metals are not recovered. In Europe, the mineral fraction of incinerator bottom ash (MIBA), i.e. BA after metal sorting and natural ageing (Neuwahl et al. 2019), is in some countries such as Denmark and Finland, reused for construction purposes outside landfills (Blasenbauer et al. 2020). However, most of the MIBA is used for the construction within landfills, e.g. in Sweden, or is even considered hazardous waste and is directly landfilled, i.e. Switzerland (Blasenbauer et al. 2020). As the supply of landfills is limited, alternative handling of the MIBA is needed. The restrictive handling of MIBA is due to the potential risk of metal leaching (Karlfeldt Fedje et al. 2021). Extracting these metals could enable recovery and safer reuse. However, as the metals remaining in MIBA are chemically bound, the recovery is challenging. Neither thermal nor hydrometallurgical processes are suitable due to the presence of unwanted elements such as Cl, Si and Al (Astrup et al. 2016) or the significant handling and purification of water and leaching agents required (Karlfeldt Fedje et al. 2021; Šyc et al. 2020). However, hydrometallurgical methods are used in a few places to recover Zn from fly ash, which has higher metal contents compared to BA (Karlfeldt Fedje and Andersson 2020; Weibel et al. 2017). Additionally, the FA generation is smaller and thus the need for leaching agents is lower.

An alternative metal recovery method could be phytoextraction. Phytoextraction uses plants that can grow in metal-rich conditions and absorb and store the metals in their plant tissues (Farooqi et al. 2022; Grzegórska et al. 2020). Historically, phytoextraction has been used to clean soil by removing metals and/or other pollutants, and in recent decades, the method has received more attention (Ghori et al. 2016; Grzegórska et al. 2020). Plant-based techniques to remediate metal-contaminated soils have limitations, such as the climate zone and cultivation time of a specific plant, the biomass production and extraction efficiency, but they are cheaper and more environmentally friendly than other alternatives that often involve expensive and technically complicated methods such as excavation, pumping, washing or burning of the soil (Ghori et al. 2016; Hooda 2007; Sheoran et al. 2016). Traditionally, phytoextraction has focused on remediating the soil material itself, but there are examples where the extracted metals are recycled from the harvested plants and reintroduced into society (Dinh et al. 2022; Han et al. 2018; Simonnot et al. 2018; Vaughan et al. 2017). Since MIBA is continuously generated and generally contains higher amounts of more valuable and/or toxic elements than contaminated sites, the potential for using phytoextraction for metal recovery on this material is theoretically high. However, knowledge about cultivating in MIBA is limited. To the authors’ knowledge, there are only two scientific papers published on the phytoextraction of MIBA (Karlfeldt Fedje et al. 2021; Rosenkranz et al. 2017), so more research is needed. Both papers conclude that cultivation in MIBA is possible, but better knowledge concerning the specific circumstances of MIBA cultivation is crucial.

The need for alternative handling of MIBA is urgent and, in this study, the potential for phytoextraction is studied. Based on a review of the published literature on the phytoextraction of metals from mainly contaminated soil, this work aims to evaluate the following research questions:

- What specific challenges are associated with cultivation in MIBA?
- Is there any economic potential in phytoextraction from MIBA?
- Can plants suitable for phytoextraction from MIBA be identified from the literature?

## Metal content and economic potential in MIBA

When the most common incineration technique of grate-firing is used, approximately 20 wt% of the incinerated waste ends up as BA, which is about 80% of the total ash amount (Brunner and Rechberger 2015). The BA is usually cooled by passing it through a water bath; afterwards, the ash is stored outside in heaps, and metal pieces are sorted out. This storage technique results in the ageing or natural weathering of the material, which reduces leaching from the ash due to carbonation, wherein alkaline compounds such as Ca(OH)_2_ in the ash absorb CO_2_ from the air to form carbonates (Freyssinet et al. 2002). The carbonation process decreases pH to slightly alkaline and transforms some metals into less soluble species (Arickx et al. 2007; Astrup et al. 2016). After this procedure, the BA is referred to as MIBA to distinguish fresh BA from processed BA (Blasenbauer et al. 2020).

The elemental content in MIBA naturally differs between WtE plants due to the waste composition and the incineration process used. In this study, MIBA samples from nine Nordic WtE plants (18 total samples (Wahlström et al. 2022)) were used (Table 1), and the variation between the plants was limited for most elements. The major elements (Si, Al, Ca, Fe, K, Mg, Mn, Na, P and Ti), calculated as oxides, account for nearly 90 wt% of the MIBA. They constitute the ash matrix itself and are in general strongly bound in various minerals such as feldspars (e.g. NaAlSi_3_O_8_ and CaAl_2_Si2O_8_), quartz (SiO_2_), calcite (CaCO_3_) and magnetite (Fe_2_O_3_) (Tiberg et al. 2021). However, this review focuses on the minor elements Cu, Zn, Pb, Ni and Co. These were primarily chosen based on their presence in MIBA, the available literature regarding phytoextraction potential and the societal demand. In addition, the metals’ economic potential in MIBA and their potential toxicity were to some extent considered.

**Table 1:** Average content, median and standard deviation for 18 Nordic MIBA samples

Major elements calculated as oxides	Average	Median [% DS]	Standard deviation
SiO_2_	38.7	40.6	9.55
Al_2_O_3_	9.77	10.2	1.27
CaO	15.0	15.0	1.97
Fe_2_O_3_	14.4	14.9	1.84
K_2_O	1.26	1.33	0.211
MgO	1.89	1.88	0.227
MnO	0.174	0.164	0.0351
Na_2_O	2.92	2.71	0.676
P_2_O_5_	1.01	0.949	0.213
TiO_2_	1.34	1.24	0.277
Minor and trace elements		[mg/kgDS]	
As	25.3	25.5	7.26
Ba	2.110	2.030	553
Be	1.94	2.00	1.12
Cd	5.18	4.50	2.70
Co	116	80.5	104
Cr	758	722	215
Cu	3.760	3.390	1.720
Hg	0.597	0.0500	0.0222
Mo	32.2	20.0	38.0
Nb	12.9	12.5	2.81
Ni	300	234	206
Pb	864	845	368
S	4.880	5.100	1.750
Sb	82.6	80.5	20.4
Sc	4.11	4.00	0.832
Sn	152	153	40.2
Sr	365	345	64.4
Tl	0.157	0.167	0.0339
U	2.055	2.00	0.416
V	51.9	53.5	8.70
W	59.8	62.5	31.6
Y	14.2	12.5	4.88
Zn	4.015	3.670	995
Zr	226	213	53.8

Major elements are calculated as oxides and given in wt% DS (dry substance), while minor elements are shown in milligrams/kilogram of DS. Data used with permission from the authors of Wahlström et al. (2022)

The metals Co, Cu, Ni, Pb and Zn are widely used in society, with annual mining production ranging between 0.19 (Co) and 22 (Cu) Mt (The U.S. Geological Survey 2023a, b, c, d, e). In terms of production, Cu and Zn are recognised as some of the most used metals worldwide, following Fe and Al. Like the other metals, Ni was selected for its many areas of application and because it is one of the most resource-intensive metals to produce and emits large quantities of CO_2eq_ (Norgate et al. 2007). Pb differs from many other metals since its production is decreasing (The U.S. Geological Survey 2023 d). This is a result of lower demand in areas other than battery use, which is likely due to its toxic properties as well as its environmental impact in terms of contaminating fresh water and marine ecosystems during mining (Tao et al. 2019). The demand for Co increases every year due to its use in rechargeable batteries, which is important in the electrification of society and Co is on the EU’s list of critical raw materials (Geological Survey of Sweden 2020; The U.S. Geological Survey 2023a). The annual production of Co in 2022 was 0.19 Mt and most of it was mined in the Democratic Republic of the Congo (The U.S. Geological Survey 2023a). This makes the supply vulnerable as the region is politically unstable (Gulley 2022). Additionally, Co has, out of the five selected metals, the lowest recycling rate, i.e. 24%, while Cu has 32% and the other three, i.e. Ni, Pb and Zn, have a recovery rate of around 60% (The U.S. Geological Survey 2023a, b, c, d, e).

Due to the limited literature on metal recovery from MIBA, the economic potential is challenging to predict. However, to achieve a theoretical economic value of recovering metals from MIBA, the total contents (Table 1) for selected elements (Co, Cu, Ni, Pb and Zn) were multiplied by the annual MIBA production in Sweden or the EU (Blasenbauer et al. 2020) and along with the average metal prices for the years 2018 to 2022 sourced from the metal market Die Bundesanstalt für Geowissenschaften und Rohstoffe (BGR) (Bastian and Kern 2023). The metal prices for the five metals of interest, along with their calculated potential values in MIBA, are given in Table 2. Co and Ni have the highest value per kilogram, but the highest value in MIBA is Cu and Zn due to their higher content, with potential values of 28.5 and 11.8 million euros in Swedish MIBA, respectively. From only these five elements, the potential annual value of Swedish MIBA is 55 million euros, and the corresponding value for European MIBA is almost 1 billion euros. It is important to note that this is the maximum theoretical economic value since metal extraction is not complete, refining elements into high-quality metals will cost money and not all MIBA is suitable for recovery. However, these approximate calculations still show the opportunities this material represents. For comparison, the Cu content in the usable ore from Aitik, the largest Swedish Cu mine, was 0.24% in 2020, which is lower than the average content in MIBA (0.38%, Table 1) (Karlsson 2020). Corresponding values for other elements present in MIBA, such as Ti, V and Sn, are given in Table S1 in the Supplementary Information section. Even though metal values are important when evaluating the phytoextraction of MIBA, the fact that MIBA itself will contain lower amounts of less-mobile metals after treatment opens new use options such as construction purposes outside landfills, which could reduce the need for virgin materials and avoid landfilling.

**Table 2:** Metal prices (BGR (Bastian and Kern 2023)) and theoretical annual values for Co, Cu, Ni, Pb and Zn in Swedish and European MIBA

Metal	Metal value [Euro/tonne]	Potential annual value of Swedish MIBA [K€]^1^	Potential annual value of EU MIBA [K€]^1^
Co	52	6000	110,000
Cu	7.6	28,500	513,000
Ni	18	5300	95,400
Pb	2.1	1900	34,200
Zn	2.9	11,800	212,400

^1^(Me content in MIBA) × (Me price) × (Amount MIBA/year in Sweden or EU)

## Challenges with MIBA concerning phytoextraction

**Fig. 1:**
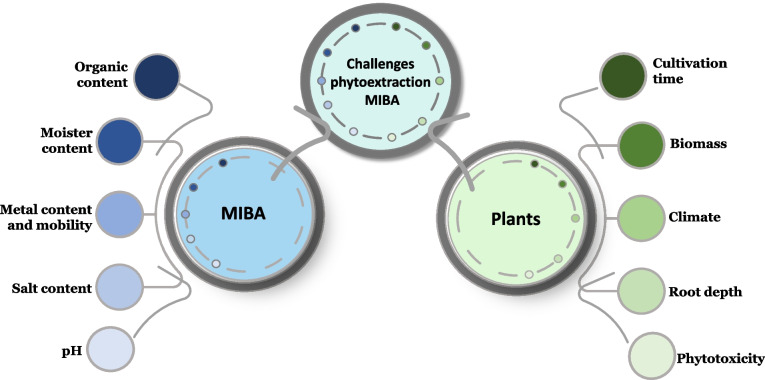
Overview of the important challenges facing cultivation in MIBA

MIBA has a high metal content, making it highly interesting for phytoextraction. The two published studies on cultivation in MIBA show that the metal content in the plants can be significant, but most of the metals are still present in the material (Karlfeldt Fedje et al. 2021; Rosenkranz et al. 2017). The mobility of metal ions in MIBA is generally low, so the full phytoextraction potential is unknown (Karlfeldt Fedje et al. 2021). Given the novelty of this topic, examining the general challenges of phytoextraction and comparing MIBA with conventional soil can assist in assessing its feasibility for cultivation and metal extraction. In addition to the general drawbacks of phytoextraction, such as the cultivation time and low biomass for many hyperaccumulators, cultivation in MIBA presents several challenges compared to soil. The most important ones are highlighted in Fig. 1. The following sections outline key differences between MIBA and soil and identify the main challenges associated with phytoextraction in MIBA. Furthermore, several strategies for improvement are examined.

The main components of MIBA, e.g. Al, Si, Fe and Ca, are similar to those of the earth’s crust and mineral soil (Haynes 2016; Karlfeldt Fedje et al. 2021). The extent of the similarities or the differences between soil and MIBA varies with the type of soil, but the high metal content in MIBA is considered a significant distinction. This is likely the most challenging property, as it can cause phytotoxicity; however, this is also why MIBA could act as a secondary raw material.

The particle size distribution in MIBA shows a higher fraction of large particles, influencing its water- and nutrient-holding capacities (Karlfeldt Fedje et al. 2021). It has been reported that MIBA cultivation needs more water compared to conventional cultivation, which is likely due to the higher fraction of large particles, i.e. more draining material (Karlfeldt Fedje et al. 2021) or the high salt content (Rosenkranz et al. 2017). Insufficient water can hinder both plant growth and metal uptake (Moray et al. 2016; Pandian and Karthik 2022; Rosenkranz et al. 2017; Sheoran et al. 2016). Additionally, MIBA contains minimal organic material and has a low N content (Astrup et al. 2016; Karlfeldt Fedje et al. 2021; Rosenkranz et al. 2017). This is naturally due to the incineration process where organic C in the waste is transferred mainly to CO_2_, while N compounds are transferred to NO_x_. Organic content in soil varies but is typically higher than the 0.2–5% found in MIBA (Astrup et al. 2016). Low content of organic matter can limit plant growth, but fertilizers can mitigate this (Karlfeldt Fedje et al. 2021; Sheoran et al. 2016).

The incineration process also gives MIBA a high pH, which affects the plant selection, in addition to metal availability. In soil, the metals’ bioavailability generally increases with lower pH (Sheoran et al. 2016). Most Swedish soils have a pH just below 7 (Stendahl 2021), whereas fresh BA often has pH levels around 11, which naturally decreases to about 8–9.5 in the ageing process (Arm 2006; Astrup et al. 2016) which occurs within the first months of storage. A longer storage period of up to 2 years does not result in lower pH (Dou et al. 2017). However, a better supply of air, which can come from mixing the ash heaps, might improve the reactions and result in somewhat lower pH values. The addition of fertilizers or acid can also decrease the pH of MIBA (Rosenkranz et al. 2017; Sheoran et al. 2016).

MIBA is more compact than ordinary soil, which can affect root growth (Karlfeldt Fedje et al. 2021). The root growth influences the depth and density of the roots, which in turn affects how much metal the roots can accumulate (Sheoran et al. 2016). The root density naturally decreases with depth, meaning the MIBA layer cannot be too deep for efficient extraction. A general consequence of this is the need for large land areas, and as MIBA is often generated in urban areas, the cost of land can be a significant expense. However, the co-cultivation of different types of plants not only provides variation in plant structure and taxonomy but also contributes to variation in root depth and density (Sheoran et al. 2016). Studies on rain gardens irrigated with stormwater have shown that structurally varying cultivation provides better results compared to single-plant cultivation (Yuan et al. 2017). This was due to better moisture-holding capacity caused by greater variations in root depths and pore sizes in the soil material. A recent study on establishing a rain garden with a combination of plants and different sorption filter materials such as MIBA to remediate polluted stormwater shows the potential for MIBA cultivation (Johansson et al. 2024). The initial results show a high reduction of pollutants such as metals like Ni and Zn in the effluent water, but it is not yet clear how much the plants extract. However, the plants grew just as well in the MIBA filters as in the control filter with ordinary soil.

Phytoextraction as a remediation technique is cost-efficient and sustainable (Ghori et al. 2016; He et al. 2023), but it faces several challenges. The risk of phytotoxicity is a key limiting factor for phytoextraction, which naturally influences the choice of plants (Rascio and Navari-Izzo 2011; Sheoran et al. 2016). Using plants that are tolerant to extreme metal concentrations is crucial. Many plants that have this tolerance and extraordinary metal uptake ability are called hyperaccumulators, and they often have slow growth rates and low biomass (Chen et al. 2004; Dhiman et al. 2016; Greger and Landberg 1999; Kumar et al. 1995; Sheoran et al. 2016). The plants’ biomass combined with the accumulated metal concentration determine the total metal extraction; therefore, low biomass results in low metal extraction (Sheoran et al. 2016). Additionally, the plant should survive in the climate zone of interest. The Nordic climate is an obstacle for vegetation because few hyperaccumulators thrive there (Karlfeldt Fedje et al. 2021). Besides these challenges, the plants’ potential for phytoextraction can vary greatly due to structural differences in plants as well as geographical, chemical and physiological conditions (Asgari Lajayer et al. 2019; Ghori et al. 2016; Karlfeldt Fedje et al. 2021).

Certain measures can be taken to improve the cultivation potential in MIBA. For instance, a fertilizer created from sewage sludge mixed with biomass and coal ash led to increased metal availability and plant growth due to the higher N and organic C content (Antonkiewicz et al. 2022). This application also counteracted wind erosion, which otherwise can be an issue if the ash material is too dry (Neuwahl et al. 2019). The addition of microorganisms such as arbuscular mycorrhiza fungi (AMF) may improve metal uptake (Cao et al. 2008; Firdaus-e-Bareen et al. 2012; Manzoor et al. 2019; Ważny et al. 2021). Earthworms can further enhance the bioavailability and root metal uptake by producing carboxylic compounds (Firdaus-e-Bareen et al. 2012; Santana et al. 2019; Sheoran et al. 2016). Chelating agents such as EDTA can improve metal solubility and transport in plants (Hasan et al. 2019; Kanwal et al. 2014). However, EDTA is environmentally persistent and potentially toxic, so dosage must be controlled (Guo et al. 2015). Combining chelating agents, such as EDTA with biodegradable alternatives such as citric or jasmonic acid, has shown promising results, enhancing plant growth and metal extraction while reducing the negative effects of high concentrations of EDTA (Ibrahim et al. 2022; Saleem et al. 2020b). Additionally, jasmonic acid can improve plant resistance to drought, salinity and metal toxicity, which are all considered challenges in MIBA. Research on jasmonic acid in phytoextraction is limited, but it shows potential.

## Potential plants for the phytoextraction of selected metals

The metals selected in this study are admissible not only for their amount in MIBA or the interest in recovering them, but also for being associated with several metal-accumulating plants, specifically those on two elaborate lists of known accumulators (Reeves et al. 2018; Wikipedia 2024). The review was focused on scientific papers but also reports and websites from established publishers like Royal Botanic Gardens, Kew, were studied to broaden the amount of available literature. In addition to providing information about the plants’ phytoextraction potential, their relevance was determined based on the following key factors:

- Firstly, only terrestrial plants were considered applicable to be grown in MIBA; grass plants were not selected due to their low biomass.
- Secondly, they needed to be able to accumulate a significant amount of metal due to the high content of various metal compounds in MIBA, commonly referred to ashyperaccumulators. The term hyperaccumulator was first introduced by Jaffré et al. (1976) and there is no strict definition of what is considered “significant” or “hyperaccumulator” in the literature, but here it is roughly based on a common definition for a hyperaccumulating plant put forth by Reeves et al. (2018). This definition includes threshold values for metal uptake in dry-weight foliar tissue. The values are > 300 mg/kg of Co and Cu, > 1000 mg/kg of Ni and Pb and > 3000 mg/kg of Zn. These are to be considered reference values and not determining factors in this work.
- Thirdly, climate conditions were considered since only plants that can grow in a Nordic climate are of interest. Generally, this means that plants with distribution in the temperate biome, which includes the southern parts of the Northern countries, are considered.

In addition to the key factors above, some further aspects were considered when the plants were determined. Those included that there should be more than one published study on a specific plant and that rare plants with very limited distribution of growing sites were not included. Furthermore, as this study is the first review on this specific topic and aims to identify the type of plants rather than evaluate the differences between hybrids and clones of specific species, such studies were also excluded. During this review, more than 100 different plants were considered and investigated as feasible for MIBA phytoextraction. Based on the criteria above, the following 13 plants were identified as the most interesting for extracting the chosen metals from MIBA. After each plant’s name, the metals discussed in the literature, are given:

- *Brassica juncea* (L.) Czern—Co, Ni Pb, Zn
- *Brassica napus* L.—Cu, Ni, Zn, Pb
- *Arabidopsis halleri* (L.) O’Kane & Al-Shehbaz—Zn
- *Bornmuellera tymphaea* (Hausskn.) *Hausskn.*—Ni
- *Cochlearia aucheri* var. minor Boiss—Ni
- *Noccaea caerulescens* (J.Presl & C.Presl) F.K.Mey—Ni, Pb, Zn
- *Noccaea goesingensis* (Halácsy) F.K.Mey—Co, Ni, Zn
- *Linum usitatissimum* L.—Cu, Pb, Zn
- *Salix alba* L.—Co, Cu, Ni, Pb, Zn
- *Salix viminalis* L.—Co, Cu, Ni, Pb, Zn
- *Sesbania drummondii* (Rydb.) Cory—Cu, Ni, Pb, Zn
- *Viola lutea* subsp. *calaminaria* (Ging.) Nauenb.—Pb, Zn
- *Viola lutea* var. *westfalica* A.A.H. Schulz—Pb, Zn

The species below belong to the Brassicaceae family, and they are all perennials except for *B. juncea and B. napus*, which are annuals [94], [97], [101], [109], [111], [114]. *B. juncea*, known as Indian mustard, and *B. napus*, known as rapeseed, are similar in many ways. They are distributed worldwide, including in the Nordic regions (Dhiman et al. 2016; Royal Botanic Gardens 2023a; SLU Swedish Species Information Centre 2024a, b). They are also fast-growing, have medium biomass, can be harvested frequently (Editors of Encyclopaedia Britannica 2024, 2023; Kumar et al. 1995) and can grow in soils with pH > 6 (Ariyakanon 2003). In soil, *B. juncea* and *B. napus* have been shown to accumulate high levels of Zn and Pb (Jiang et al. 2000; Kumar et al. 1995; Marchiol et al. 2004; Ariyakanon 2003; Weerakoon and Somaratne 2009). One study using *B. juncea* also showed it accumulated a high level of Co (Weerakoon and Somaratne 2009). Both plants have been proven to grow in MIBA and efficiently extract Zn (Karlfeldt Fedje et al. 2021; Rosenkranz et al. 2017). In the study by Karlfeldt Fedje et al. (Karlfeldt Fedje et al. 2021), *B. napus* extracted many other metals besides Zn, such as Cu, Pb, Ni and Co. The latter two were accumulated to a lower degree however. In the study by Rosenkranz et al. (2017), *B. napus* and *B. juncea* showed limited uptake of Ni and Cu, making Zn the only metal accumulated to a significant degree, but few metals were measured. In both studies on MIBA, these plants were found to be relatively tolerant to a reduction in biomass due to high metal content. *A. halleri* is a wintergreen species and thrives in several places in Europe (Honjo and Kudoh 2019; Royal Botanic Gardens 2023b), but no distribution in Sweden has been reported (SLU Swedish Species Information Centre 2024c). It is an efficient Zn accumulator (Reeves 2006) but it has low biomass. Additionally, it can grow in metalliferous or non-metalliferous soils (Bert et al. 2002). *B. tymphaea* and *C. aucheri* are Ni accumulators (Chardot et al. 2005; Reeves 2006; Reeves et al. 1983) that grow in Southern Europe (Chardot et al. 2005; Govaerts et al. 2021), but *B. tymphaea* could possibly be cultivated elsewhere if the climate is similar to its native habitat. Both can grow on serpentine soils (Chardot et al. 2005; Reeves 1988). Additionally, *C. aucheri* has low biomass, while *B. tymphaea* has medium biomass. *N. caerulescens*, known as alpine pennycress, and *N. goesingensis* are described as commonly growing in metal-rich serpentine soils and metal mine waste at pH levels > 6 and they efficiently accumulate Zn and Ni (Brown et al. 1995; Chardot et al. 2005; Mandáková et al. 2015; Reeves 2006; Reeves and Baker 1984; Reeves and Brooks 1983; Robinson et al. 1998; Ważny et al. 2021). Additionally, *N. caerulescens* accumulates Pb in large amounts (Reeves 2006; Robinson et al. 1998) and *N. goesingensis* accumulated a high level of Co in one study (Reeves and Baker 1984). *N. caerulescens* grows throughout Europe, including Scandinavia (Mandáková et al. 2015; Royal Botanic Gardens 2023c; SLU Swedish Species Information Centre 2023), while *N. goesingensis* mainly grows in the temperate biome limited to a few countries in Central to Southeastern Europe (Royal Botanic Gardens 2023 d; Ważny et al. 2021; Wenzel and Jockwer 1999). Both plants have relatively low biomass (Svedberg and Feildberg 2000). A study including *N. caerulescens* growing in metal mine waste showed that the biomass was not significantly affected by high dosages of metal (Robinson et al. 1998).

The species below belong to different families and are all perennial plants, except for* L. usitatissimum. L. usitatissimum*, commonly known as flax, is a relatively fast-growing plant with medium biomass that can reach heights of up to 1 m (Royal Botanic Gardens 2023e; Saleem et al. 2020a). It can grow in various soil types in both cold and warm climates. *L. usitatissimum* is mainly annual, but some varieties are perennials. This plant has been shown to accumulate Cu, Pb and Zn (Hosman et al. 2017; Saleem et al. 2020c). Cu was efficiently extracted and distributed equally between the roots and shoots, but biomass reduction was noticed with Cu concentrations > 400 mg/kg in the soil (Saleem et al. 2020c). *S. alba*, known as osier willow, and *S. viminalis*, known as white willow, are associated with wet habitats such as wetlands, but they can tolerate different soil types as long as the roots have access to water (Durrant et al. 2016; Go Botany 2024). *S. viminalis* is a shrub or small tree reaching up to 10 m (Arbolapp n.d.; Go Botany 2024), whereas *S. alba* can reach heights of up to 30 m and a diameter > 1 m (Durrant et al. 2016). Neither have exhibited extraordinary accumulation values for any of the selected metals (Hammer et al. 2003; Keller and Hammer 2005; Mleczek et al. 2018, 2010, 2009) but they can still be relevant for MIBA phytoextraction due to their fast growth and high biomass as well their worldwide distribution, which includes the Nordic regions. They also provide the opportunity of frequent harvests (Arbolapp n.d.; Durrant et al. 2016; Go Botany 2024; Pulford and Watson 2003; Royal Botanic Gardens 2024a, b). Several studies have shown that both *S. viminalis* and *S. alba* can accumulate Zn and many other metals such as Co, Cu, Ni and Pb, but in limited concentrations independently of pollution level (Hammer et al. 2003; Mleczek et al. 2018, 2010, 2009). In one of the studies, *S. viminalis* was cultivated in a mining sludge with a high metal content (Mleczek et al. 2018) that had a composition similar to MIBA. The accumulated content was higher in the plants growing in the polluted area compared to the reference soil, but uptake was still limited when compared to the threshold values considered in this work. It has been suggested that some elements, such as Fe and Ca, can block the uptake of other elements, but more research is required (Mleczek et al. 2018). The significant variabilities between Salix species and hybrids, as well as the wide genetic variation and other complex factors, such as the interplay between different metals, have also been discussed as reasons for the relatively low extraction efficiency of Salix (Mleczek et al. 2010, 2009; Pulford and Watson 2003). However, the studies by Mleczek et al. (2018, 2010, 2009) concluded that the high biomass yield counterbalanced the low extraction efficiencies, so Salix was deemed to be sufficient for phytoremediation processes. Additionally, high biomass is connected with thicker and deeper roots, which is favourable when cultivating in dense MIBA. *S. drummondii*, or Rattlebush, has demonstrated the ability to accumulate several metals to varying degrees, especially Cu and Pb, whose accumulations were particularly high, followed by Zn and Ni (Israr et al. 2011; Shivendra V. Sahi et al. 2002). *S. drummondii* can grow in substrates with various pH levels, but it accumulates lower amounts as pH increases (up to 7.7) (Shivendra V. Sahi et al. 2002). This perennial plant belonging to the Fabaceae family has high biomass, can grow quickly up to 4 m in height and is mainly distributed throughout southern North America (Jenkins 2020; Shivendra V. Sahi et al. 2002; Temperate Plants Database, n.d.). Since it grows within the warmer zone of temperate climates, it might be less suitable for Nordic regions. Like Salix, it prefers moist soil, possibly making cultivation in MIBA challenging. It is also worth noting that the seeds are poisonous if ingested, so one might have to limit wildlife from entering the area. *V. calaminaria* and *V. westfalica* might be suitable for MIBA phytoextraction in the north since they tolerate a cold climate and thrive in metal-rich environments. However, they are limited to a few areas in Germany and Belgium (Bizoux et al. 2004; Bothe and Słomka 2017; Jedrzejczyk-Korycinska et al. 2002), have low biomass and prefer moist soil, characteristics which might decrease the phytoextraction potential when grown in MIBA. These perennial flowers belong to the Violaceae family of flowering plants and are sometimes referred to as zinc violets. They are especially efficient at accumulating Zn, but their accumulation of Pb has also been reported (Bizoux et al. 2004; Jedrzejczyk-Korycinska et al. 2002). In the areas where these cultivation trials have been carried out, the soil pH ranged between 6 and 8.

A summary of the most extractable metals from soil and their amounts in roots and shoots, as well as the total biomass of the selected species, is shown in Table 3. It is important to note that the data on the species is not always the same since the researched references focus on phytoremediation and thus vary in terms of what is included about the plant itself. The extracted levels vary significantly due to factors such as soil properties, cultivation methods, degree of contamination and whether the contaminants are natural or added. Consequently, the varying data makes it challenging to determine the reasons for specific experimental outcomes. The focus here is solely on the accumulated concentrations from successful trials to demonstrate the suitability of the plants. The approximate biomass for each plant is also shown since high biomass is beneficial. Low biomass (L) generally refers to small flowering plants with short and/or thin stems, medium biomass (M) may signify either taller or thicker flowering plants along with bushes or smaller shrubs, while high biomass (H) represents trees or large shrubs.

**Table 3:** Approximate biomass (low, medium, high) and examples of accumulated concentrations of metals in different plant types cultivated in soil. Uptake is calculated on 100% dry substance (DS)

Species	Biomass (L, M, H)	Metal and original soil content (mg/kg DS)	Uptake in plant, root (R) or above ground (AG) (mg/kg)	Comment	Reference
*B. juncea*	M	Co 16,700	9800–18,500**	Field trial (approx. 3 months) in contaminated soil. Lowest and highest mean concentration of *B. juncea* (L.) *Cazen* during two seasons in Sri Lanka	Weerakoon and Somaratne (2009)
		Pb*	6000(R), 80 (AG)	Pot trial (< 1 month) with *B. juncea* L. var. *Megarrhiza*. The soil was spiked with 10^−3^ M of lead nitrate. This concentration slightly inhibited its growth. The AG accumulation was measured in the shoots	Jiang et al. (2000)
		Pb 625	103,500 (R), 10,300 (AG)	Pot trial (< 1 month) with Pb-spiked coarse-grained sand mixed with perlite. The AG accumulation was measured in the shoots	Kumar et al. (1995)
		Pb 11,300	3600–15,600**	See comment for Co and reference (Weerakoon and Somaratne 2009) above	Weerakoon and Somaratne (2009)
		Zn 1000	2800 (AG), 1800 (R)	Pot trial (approx. 2 months) with *B. Juncea* var. *Coss* in Zn-spiked soil. Substrate pH of 6.21	Ariyakanon (2003)
		Zn 651,500	361,800–641,000**	See comment for Co and reference (Weerakoon and Somaratne 2009) above	Weerakoon and Somaratne (2009)
*B. napus*	M	Pb 625	61,200 (R), 3400 (AG)	See comment for Pb for *B. juncea* and reference (Kumar et al. 1995) above	Kumar et al. (1995)
		Zn 6685	1300 (AG), 6000 (R)	Pot trial (approx. 2 months) in contaminated soil mixed with sand. Substrate pH of 6.2. Stunted growth occurred in preliminary tests using only the contaminated soil. The AG accumulation was measured in the shoots	Marchiol et al. (2004)
*A. halleri*	L	Zn*	13,600**	From a compilation of studies	Reeves (2006)
*B. tymphaea*	M	Ni 471	5600 (AG)	Pot trial (approx. 3 months) in contaminated serpentine soil. Substrate pH slightly below 6	Chardot et al. (2005)
		Ni*	1600–31,200 (AG)	Accumulation was measured in the leaves from plants collected from several institutions	Reeves et al. (1983)
*C. aucheri*	L	Ni*	11,500–17,600**	From a compilation of studies. In serpentine soil	Reeves (1988)
*N. caerulescens*	L	Ni 471	4800 (AG)	See comment for Ni for *B. tymphaea* and reference (Chardot et al. 2005) above	Chardot et al. (2005)
		Pb*	2700**	From a compilation of studies	Reeves (2006)
		Pb 16,500	840**	Field trial with wild plants growing in metal mine wastes in France. Substrate pH of 6.4–7.7. The result is for 1-year plants. Two-year plants had much lower concentrations, perhaps due to a dilution effect over time	Robinson et al. (1998)
		Zn 38,000	11,600**	See the comment above	Robinson et al. (1998)
		Zn*	85,000 (R), 26,000 (AG)	Pot trial (> 1 month) in soil with 3160 µM of Zn. This concentration caused no visible signs of Zn toxicity. The AG accumulation was measured in the shoots	Brown et al. (1995)
		Zn*	43,700**	From a compilation of studies	Reeves (2006)
*N. goesingensis*	L	Ni (*), 4710	5000–12,400 (AG), 2310 (AG)	Measurements in the leaves of herbarium specimens growing in serpentine soils and in shoots from a pot trial (< 3 months) in serpentine soil (“NC” in the study) and serpentine seed origin. Substrate pH of 6–8	Reeves and Baker (1984)
		Zn (*), 1520	170–3800 (AG), 11,200 (AG)	Measurements in the leaves of herbarium specimens growing in serpentine or calcareous soils and in shoots from a pot trial (< 3 months) in serpentine soil with added Zn (“DMZ” in the study) and seed origin. Substrate pH of 6–8	Reeves and Baker (1984)
		Co 491	> 1390 (AG)	Pot trial (< 3 months) in serpentine soil (“NC” in the study) with pH of 6–8. Accumulation was measured in the shoots	Reeves and Baker (1984)
*L. usitatissimum*	M	Cu 600	280 (R), 820 (AG)	Pot trial (approx. 5 months) in China in Cu-spiked soil. Substrate pH of 5.6. Reduction in biomass began at lower concentrations (400 mg/kg). The AG accumulation was measured in the shoots. This harvesting interval resulted in more metal being translocated to the roots compared to earlier harvests with similar total uptake concentrations	Saleem et al. (2020c)
		Pb 704	311**	Pot trial (1–5 months) in soil spiked with Pb and Zn. Substrate pH of 7.8. Lower metal concentrations in the soil resulted in a higher removal percentage than this accumulation	Hosman et al. (2017)
		Zn 1008	256**	See the comment above	Hosman et al. (2017)
*S. alba*	H	Cu 4.3–7.48	~ 4–6(AG)	In two natural soils in Poland with low metal concentrations, one being a sandy river soil and the other an acidic clayey soil. Metal concentrations were measured in branches or shoots taken from heights of around 0.1 to 1 m	Mleczek et al. (2010, 2009)
		Ni 7.2	~ 4 (AG)	In a natural sandy river soil in Poland with low metal concentration. Metal concentrations were measured in branches taken from heights of 0.1 to 1 m	Mleczek et al. (2009)
		Pb 6.10–7.2	~ 4–7 (AG)	See comment for Cu and reference (Mleczek et al. 2010, 2009) above	Mleczek et al. (2010, 2009)
		Zn 21.5–31.30	~ 60–100 (AG)	See comment for Cu and reference (Mleczek et al. 2010, 2009) above	Mleczek et al. (2010, 2009)
*S. viminalis*	H	Cu 7.48	~ 6 (AG)	In a natural acidic clayey soil in Poland with a low metal concentration. Metal concentrations were measured in shoots taken from heights of 0.95 to 1.05 m	Mleczek et al. (2010)
		Cu 5291	182 (AG)	Cultivation in an extremely polluted mining sludge. Metal concentrations were measured in the leaves. Substrate pH > 8	Mleczek et al. (2018)
		Pb 6.10	~ 4 (AG)	See comment for Cu and reference (Mleczek et al. 2010)	Mleczek et al. (2010)
		Pb 6228	333 (R)	Cultivation in extremely polluted mining sludge. This is the total accumulation in the roots (lateral and primary roots). Substrate pH > 8	Mleczek et al. (2018)
		Zn 650	390–1100 (AG), 70–200 (AG)	Grown in calcareous soil (pH 7.3). Measurements for 2 years. This result accounts for uptake in the leaves and the stems, although most metals were found in the leaves. The concentration became lower with time, but biomass increased	Hammer et al. (2003)
		Zn 31.30	~ 55 (AG)	See comment for Cu and reference (Mleczek et al. 2010)	Mleczek et al. (2010)
		Zn 1158	1700–2700 (AG), 430–570 (AG)	Grown in acidic soil (pH 5.2). Measurements for 5 years. This result accounts for uptake in the leaves and the stems, where most were found in the leaves. The concentration generally became lower with time	Hammer et al. (2003)
		Zn 6808	806 (AG)	See comment for Cu and reference (Mleczek et al. 2018)	Mleczek et al. (2018)
*S. drummondii*	H	Cu*	9800–15,600 (R), 200–500 (AG)	Pot trial (approx. 1 month) in soil receiving varying amounts of several metals (Pb, Cu, Ni, Zn). Substrate pH of 5.6. The AG accumulation was measured in the shoots. The highest result came from the Cu + Ni treatment	Israr et al. (2011)
		Ni*	480–2100 (R), 100–400 (AG)	Same as above, except the highest accumulation was achieved when the soil only was treated with Ni	Israr et al. (2011)
		Pb*	32,700–64,800 (R)	Same as above, except the highest accumulation was achieved with the Pb + Cu treatment	Israr et al. (2011)
		Pb*	~ 60,000 (R), ~ 60,000 (AG)	Pot trials (< 1 month) in soil mixed with varying concentrations of lead nitrate and EDTA. This root accumulation refers to when 500 mg/L Pb was added, and the AG accumulation (in the shoots) when 1000 mg/L Pb and 100 µM EDTA were added. Varying pH levels (3.7 to 7.7) were tested. A higher pH led to poorer accumulation	Shivendra V. Sahi et al. (2002)
		Zn*	5600–10,400 (R), 780–1870 (AG)	See comment for Cu and reference (Israr et al. 2011) above, though the highest result was when the soil was only treated with Zn	Israr et al. (2011)
*V. calaminaria*	L	Pb 1000	< 10 (AG), 180 (R)	Pot trial (approx. 4 months) in soil spiked with Zn and Pb. Substrate pH of 7–8. The AG accumulation was measured in the shoots	Jedrzejczyk-Korycinska et al. (2002)
		Pb*	9300**	Measurements in species found in various contaminated soils in Belgium. Substrate pH between 6 and 7	Bizoux et al. (2004)
		Zn 10,000	1300 (AG), 4000 (R)	See comment for Pb and reference (Jedrzejczyk-Korycinska et al. 2002) above	Jedrzejczyk-Korycinska et al. (2002)
		Zn*	11,900**	See comment for Pb and reference (Bizoux et al. 2004) above	Bizoux et al. (2004)
*V. westfalica*	L	Pb 1000	55 (AG), 550 (R)	See comment for Pb and reference (Jedrzejczyk-Korycinska et al. 2002) above	Jedrzejczyk-Korycinska et al. (2002)
		Zn 10,000	1100 (AG), 2800 (R)	See comment for Pb and reference (Jedrzejczyk-Korycinska et al. 2002) above	Jedrzejczyk-Korycinska et al. (2002)

*Original concentration in the soil not specified per dry weight of soil

**Not specified if parts above ground or roots

Without real and time-consuming experiments, it is hard to evaluate the best choice for phytoextracting several metals from MIBA since there are many cultivation parameters to consider based on the specific challenges identified in Fig. 1. Some parameters are, however, extremely important, such as biomass, extraction capacity, the number of different metals, whether the plant is annual or perennial, harvesting options (root or leaves) and metal recyclability. Based on this, Table 4 shows the qualitative multi-criteria analyses (MCA) performed on the selected plants to identify the most interesting plants for real MIBA extraction experiments.

**Table 4:** Qualitative MCA of plants evaluated for phytoextracting MIBA

Plant	Biomass	Extraction capacity^1^	Metal diversity	Perennial (3) or annual (1)	Metal accumulation above ground (3) or roots (1)	Sum
*S. drummondii*	3	3	3 (Cu, Ni, Pb, Zn)	3	2	**14**
*S. alba*	3	1	3 (Cu, Ni, Pb, Zn)	3	3	**13**
*S. viminalis*	3	1	3 (Cu, Pb, Zn)	3	3	**13**
*N. caerulescens*	1	3	3 (Ni, Pb, Zn)	3	2	**12**
*B. juncea*	2	3	3 (Co, Ni, Pb, Zn)	1	2	**11**
*B. napus*	2	3	3 (Cu, Ni, Pb, Zn)	1	2	**11**
*B. tymphaea*	2	2	1 (Ni)	3	3	**11**
*N. goesingensis*	1	2	3 (Co, Ni, Zn)	3	2	**11**
*A. halleri*	1	3	1 (Zn)	3	2	**10**
*C. aucheri*	1	3	1 (Ni)	3	2	**10**
*V. calaminaria*	1	2	2 (Pb, Zn)	3	2	**10**
*V. westfalica*	1	2	2 (Pb, Zn)	3	2	**10**
*L. usitatissimum*	2	1	3 (Cu, Pb, Zn)	1	2	**9**

A rating between 1 and 3 is given for each plant and parameter, where 3 represents the best phytoextraction properties. For each parameter, a 3 signifies high biomass, high extraction capacity (> 10,000 mg/kg DS), metal diversity (extraction of many different metals), the plant is perennial and accumulation occurs in parts above ground, signifying easier harvesting

^1^1 = < 1000 mg/kg DS; 2 = 1000–10,000 mg/kg DS; 3 = > 10,000 mg/kg DS

Based on this qualitative evaluation of the selected plants, *S. drummondii* received the highest score followed by *S. alba* and *S. viminalis*. All these plants are perennial and can extract several metals, although the extraction efficiency is low to moderate for Salix. This is compensated for by its high biomass and fast growing rate compared to other plants with higher extraction capacity. All other plants, except *S. drummondii*, have either low biomass or are annuals, which are unfavourable from remediation and management perspectives. One drawback of *S. drummondii* is that it prefers a somewhat warmer climate, while Salix is known to grow in the Nordic climate, as was discussed for each plant above. Most high-accumulating plants are mainly found in warm or tropical climates. Cultivation in greenhouses may, therefore, be relevant, and heating with low-grade waste heat, such as from WtE incineration processes, could be interesting to study. This opens up the opportunity to use a larger variety of plant types in the Nordic climate. The two published scientific phytoextraction studies on cultivation in MIBA (Karlfeldt Fedje et al. 2021; Rosenkranz et al. 2017) included *B. napus*, which has good metal extraction capabilities. However, its limited biomass and annual life cycle were seen as drawbacks. The perennials are assumed to be advantageous since they require less management and develop larger roots and greater biomass over time. However, there might be a maximum metal accumulation limit, and cultivation over several years might result in decreased concentrations, which was found to be the case for *N. caerulescens* (Robinson et al. 1998). Further studies should address metal accumulation over time for different plants when cultivated in MIBA.

Although serpentine and calcareous soils are quite different, e.g. their compositions and effect on plant growth, both share some similarities with MIBA. Serpentine soil, which is derived from weathered ultramafic rocks, typically has low macronutrient content, low organic content and elevated levels of heavy metals (Kazakou et al. 2008; Koleli et al. 2015; Lazarus et al. 2011; Rajakaruna and Boyd 2014). They are often described as rocky and shallow with a granular texture and poor moisture retention capacity. Similar properties can be found in MIBA, although the Ca to Mg ratio differs and the pH levels in serpentine soil are typically between 6 and 8, which does not quite reach the typical pH of MIBA. Calcareous soil is formed due to the accumulation of calcium carbonate (CaCO_3_) from weathered carbonate-rich rocks (Singare et al. 2022; Taalab et al. 2019). Besides the high calcium content, metal concentrations (e.g. Al, Fe, Zn) can be significant, and pH levels may vary between 7 and 9. The metal content and alkaline pH are similar to MIBA. It is otherwise characterised by a more sandy or clayey texture along with poor water holding capacity, but that is mainly due to the formation of surface crusting or sub-surface hard pans. Overall, serpentine soil is considered most comparable to MIBA, and plants thriving in this soil type would likely survive in MIBA. In this review, *N. caerulescens* and *N. goesingensis* showed that they possessed promising properties for cultivation in MIBA, but their low biomass is a drawback, as indicated in Tables 3 and 4. Similar plants with a higher biomass would be interesting to evaluate in future research.

The plants were mainly selected for their properties and potential for MIBA phytoextraction in a Nordic climate. However, the presence and economic value of the different metals in MIBA are also of interest when evaluating suitable plants. As presented previously, Cu was the metal of the five selected with the highest economic value in terms of content in MIBA, Table 2. This means that Cu should have been the most profitable to extract based on price. However, this study shows that phytoextracting Cu in a Nordic climate is limited due to the few species that can accumulate Cu, and for the most part not in any extreme concentrations. Instead, the hyperaccumulators of Cu and Co are typically found in warmer climates, particularly within certain mining regions in southcentral Africa (Baker and Brooks 1989; Van Der Ent et al. 2019). While some Ni-accumulators selected in this study grow in southern Europe or North America and were deemed suitable, there are many more Ni-accumulators to be found within the Alyssum taxa, with the majority distributed within southern Europe (Palmer et al. 2001). Co and Ni have high economic values, but their lower concentrations in MIBA reduce their overall economic value. We found that many Pb accumulators with different accumulation rates and biomass production were able to grow in the Nordic climate, but the economic value of Pb in MIBA is low. Therefore, the reasons for extracting a particular metal from MIBA need to be considered. Zinc does not have high economic value, but it is present in high amounts in MIBA and has the most accumulators that can grow in a Nordic climate. This gives phytoextracting Zn high overall potential.

Until now, the focus on phytoextraction has been to extract environmentally hazardous metals such as Pb from polluted soils. Typically, there is only one or a few polluting elements present at each site, in contrast to MIBA which has several different metals present for extraction. Most of the studied plants accumulate several of the metals of interest, Table 3, but due to differing total amounts, the presence of some metals might inhibit the uptake of others (Mleczek et al. 2018). Additionally, the plant evaluation in this review focused primarily on the parameters in Table 4, but the parameters in Fig. 1 are also important to consider. For instance, tolerance to salt could be of importance when cultivating in MIBA, but this was usually not discussed in the literature included in this work. Overall, this work shows that phytoextraction is a method that could potentially be used to treat MIBA, but real cultivation experiments are needed to evaluate how efficient this could be. In addition to thorough experiments concerning, e.g. the extraction possibilities of the suggested plants in MIBA, land area use could be an issue if this should be used on a full scale. Approximate calculations, assuming a MIBA density of 1.7 tonnes/m^3^ and a depth of 1 m, suggest that the area needed to treat all Swedish MIBA using phytoextraction is about 60 ha; the corresponding area in the EU is a bit more than 1000 ha. Assuming a phytoextraction cycle takes 10 years to achieve high enough extraction quotes, the total needed areas are 600 and 10,000 ha, respectively. In comparison, 600 ha corresponds to about 0.02% of the Swedish arable land (The Swedish Board of Agriculture (2021). However, in countries with higher population densities than Sweden, the area needed can be a challenge. Even though suitable land areas can be difficult to find, the cost, if all MIBA were to be landfilled, is likely a larger problem. Only the landfill tax, about 70 euros per tonne since January 2025 (Government Offices of Sweden 2024), would correspond to about 74 million euros per year in Sweden. The landfill tax varies within the EU, but assuming a uniform tax would result in a cost of 1300 million euros per year. Consequently, the need for research on sustainable handling of MIBA is urgent, and phytoextraction offers an alternative. Additionally, if plants with high biomass are used and incinerated, not only metals could be recovered from the ash, but also the fraction of biofuel in WtE plants is increased resulting in less fossil CO_2_ emissions.

## Conclusions

In this work, phytoextraction to treat MIBA has been studied and suitable plants are suggested based on a literature review. This review provides valuable information for designing further research and the results and conclusions can be summarised in the following research questions:

- What specific challenges are associated with cultivation in MIBA?

The high metal content in MIBA is likely the most challenging property because it can cause phytotoxicity. However, this is also why MIBA could be used as a secondary raw material. The particle size distribution in MIBA shows a higher fraction of large particles, which affects both its water- and nutrient-holding capacities. MIBA is also more compact than ordinary soil, which can affect root growth and consequently the amounts of metals the roots can accumulate. Additionally, due to incineration, MIBA has a low amount of organic material and low nitrogen content as well as an alkaline pH. All these factors make phytoextraction from MIBA more complex compared to from soil. Cultivation experiments are needed to evaluate the potential effects and how to overcome them, e.g. mixing the MIBA with organic material to make it less dense and increase the organic content, choice and addition of fertilizer or other additives, and the effects from drought and heavy rainfall on water- and nutrient-holding capacities.

- Is there any economic potential in phytoextraction from MIBA?

In 2019, about 18 Mt of bottom ash from WtE was produced in the EU, including Norway and Switzerland. Theoretical calculations based on the total amounts of Co, Cu, Ni, Pb and Zn, indicate that the corresponding value is almost 1 billion euros and the indicative value for Swedish MIBA is 55 million euros. It is not realistic to expect that all metals could be extracted, but recovering part of the metals not only generates income, but the remaining metals are likely less mobile, promoting the utilisation of the residual MIBA. A worst-case scenario is landfilling the MIBA, which in Sweden would result in a cost of 74 million euros annually for the tax only. In that perspective, there is an economic potential in extracting metals from MIBA, especially as the alternative cost risks are high.

- Can plants suitable for phytoextraction from MIBA be identified from the literature?

In this review, over 100 different plants were considered and investigated to determine their potential to extract metals from MIBA through phytoextraction. Based on the outlined criteria and a qualitative MCA, the study found that among the selected plants, *S. drummondii* received the highest score, followed by *S. alba* and *S. viminalis*. All these perennial plants can extract various metals, although Salix’s extraction efficiency is low to moderate. However, this is offset by its high biomass and rapid growth rate compared to other plants with higher extraction capacity. Even though these plants have promising characteristic, this must be tested in real MIBA cultivation experiments. For instance, how do these plants survive and extract metals when cultivated in MIBA, how thick can the MIBA layer be and how closely can the plants be set to achieve optimal phytoextraction properties are important questions to evaluate before full-scale implementation.

## Supplementary Information

Below is the link to the electronic supplementary material.

ESM 1 (DOCX 27.8 KB)

## Data Availability

The data supporting the results reported in this paper can be accessed by contacting the authors.

## Declarations

All authors have approved the publication of this work.
