# Supplementary material for: Evaluating the potential of phytoextraction on waste-to-energy bottom ash—a review
Source: Environ Sci Pollut Res Int. 2025 Apr 14;32(18):11423–42. doi: 10.1007/s11356-025-36399-z (PMC12049330; doi:10.1007/s11356-025-36399-z)
Supplement: Supplementary file 1 — (DOCX 27.8 KB) [file 11356_2025_36399_MOESM1_ESM.docx]

**Supplementary information**

**Table S1**: The metal values for selected elements and the potential economic value of Swedish and European MIBA based on average content in 9 samples of Nordic MIBA [35]. The data was collected from Die Bundesanstalt für Geowissenschaften und Rohstoffe [43], the Shanghai Metals Market [133] and the London Metal Exchange [134]. The data from [43] are average values between 2018-2022, while [133] and [134] were collected on specific dates.

| **Metal** | **Metal value [Euro/kg]** | **Potential annual value Swedish MIBA [kEuro]** | **Potential annual value Europeans MIBA [kEuro]** | **Metal form and access date** | **Ref.** |
| --- | --- | --- | --- | --- | --- |
| Al | 2.2 | 114,735 | 2,065,227 | Aluminium, LME | [43] |
| As | 1.0 | 25 | 458 | Arsenic metal Accessed 2024-07-02 | [133] |
| Ca | 4.2 | 447,042 | 8,046,761 | Calcium 98.5% Accessed 2023-04-28 | [133] |
| Cd | 4.6 | 24 | 426 | 0# Cadmium Ingot Accessed 2024-07-02 | [133] |
| Co | 52 | 6,005 | 108,086 | Kobalt, LME | [43] |
| Cr | 9.7 | 7,374 | 132,731 | Chromium, SMM | [43] |
| Cu | 7.6 | 28,542 | 513,759 | Copper, LME | [43] |
| Fe | 0.9 | 91,562 | 1,648,119 | Steel Accessed 2023-03-23 | [134] |
| K | 25 | 258,289 | 4,649,198 | Potassium Accessed 2023-03-23 | [133] |
| Mg | 3.1 | 35,049 | 630,887 | Magnesium 9990 FOB CHINA Accessed 2023-03-21 | [133] |
| Mn | 2.6 | 3,526 | 63,470 | Manganese, SMM | [43] |
| Mo | 65 | 2,087 | 37,563 | Molybdenium strip Accessed 2023-03-21 | [133] |
| Na | 2.4 | 52,397 | 943,153 | Sodium Accessed 2023-03-21 | [133] |
| Nb | 92 | 1,189 | 21,402 | Niobium Accessed 2023-04-13 | [133] |
| Ni | 18 | 5,261 | 94,695 | Nickel, LME | [43] |
| Pb | 2.1 | 1,855 | 33,393 | Lead, LME | [43] |
| Sb | 21 | 1,718 | 30,930 | 0# Antimony Ingot Accessed 2024-07-02 | [133] |
| Se | 33 | 65 | 1,170 | Selenium Ingot >-99.9 % Accessed 2024-07-02 | [133] |
| Si | 2.5 | 453,865 | 8,169,574 | Silicon, SMM | [43] |
| Sn | 25 | 3,757 | 67,622 | Tin, LME | [43] |
| Ti | 16 | 126,398 | 2,275,167 | Titanium plate Accessed 2023-03-21 | [133] |
| V | 22 | 1,141 | 20,547 | Vanadium Accessed 2023-03-21 | [133] |
| W | 36 | 2,143 | 38,568 | Wolfram, Ferroalloynet | [43] |
| Zn | 2.9 | 11,763 | 211,738 | Zinc, LME | [43] |
